# Criminal of Adverse Pregnant Outcomes: A Perspective From Thyroid Hormone Disturbance Caused by SARS-CoV-2

**DOI:** 10.3389/fcimb.2021.791654

**Published:** 2022-01-03

**Authors:** Qiman Shi, Min Wu, Pei Chen, Bo Wei, Hailong Tan, Peng Huang, Shi Chang

**Affiliations:** ^1^ Department of General Surgery, Xiangya Hospital Central South University, Changsha, China; ^2^ Clinical Research Center for Thyroid Disease in Hunan Province, Changsha, China; ^3^ Hunan Provincial Engineering Research Center for Thyroid and Related Diseases Treatment Technology, Changsha, China; ^4^ National Clinical Research Center for Geriatric Disorders, Xiangya Hospital, Changsha, China

**Keywords:** COVID-19, SARS-CoV-2, adverse pregnancy outcomes, thyroid hormone, immune response

## Abstract

Nowadays, emerging evidence has shown adverse pregnancy outcomes, including preterm birth, preeclampsia, cesarean, and perinatal death, occurring in pregnant women after getting infected by severe acute respiratory syndrome coronavirus 2 (SARS-CoV-2), but the underlying mechanisms remain elusive. Thyroid hormone disturbance has been unveiled consistently in various studies. As commonly known, thyroid hormone is vital for promoting pregnancy and optimal fetal growth and development. Even mild thyroid dysfunction can cause adverse pregnancy outcomes. We explored and summarized possible mechanisms of thyroid hormone abnormality in pregnant women after coronavirus disease 2019 (COVID-19) infection and made a scientific thypothesis that adverse pregnancy outcomes can be the result of thyroid hormone disorder during COVID-19. In which case, we accentuate the importance of thyroid hormone surveillance for COVID-19-infected pregnant women.

**Graphical Abstract:**
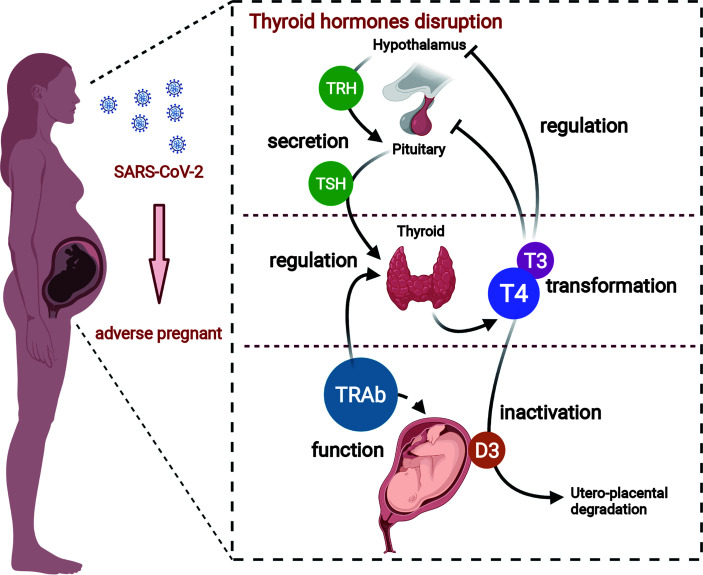
A better understanding of adverse pregnant outcomes in pregnant women with COVID-19. SARS-CoV-2 causes an over-activation of the immune response and culminates in a "cytokine storm", which, on the one hand, leads to a disturbance in maternal thyroid hormone, and on the other hand, causes dysfunction of TH transporter and deiodinase in placenta. Thus, the overall thyroid hormone disturbance in pregnant women eventually induces placenta dysfunction.

## 1 Introduction

The SARS-CoV-2 (severe acute respiratory syndrome coronavirus 2) causes coronavirus disease 2019 (COVID-19) and an ongoing severe pandemic. As of May 16, 2021, confirmed infections have amounted to 162,177,376, with casualties reaching an alarming number of 3,364,178 (https://www.who.int/emergencies/diseases/novel-coronavirus-2019). SARS-CoV-2, a novel enveloped RNA beta-coronavirus, infects host through angiotensin II-converting enzyme 2 (ACE2), a membrane-bound aminopeptidase that functions as its putative receptor predominantly expressed within type II alveolar cells of the lung. In addition to ACE2, SARS-CoV-2 requires the cellular protease, TMPRSS2, to cleave viral spike protein and facilitate fusion of viral and cellular membranes (Hoffmann et al., 2020; Zhou et al., 2020). Judging from sequencing data, SARS-CoV-2 shares more than 80% similarity with the SARS-CoV (Gralinski and Menachery, 2020; Xu et al., 2020; Zhou et al., 2020), which caused an outbreak in 2002 and shares 50% sequence similarity with Middle East respiratory syndrome (MERS) coronavirus that erupted in 2012 (Wang et al., 2020).

Previous studies have shown that pregnant women are at greater risk of morbidity and mortality due to many of the fatal viral infections, including hepatitis E virus, influenza A virus, SARS-CoV, and MERS-CoV (Rasmussen et al., 2020). Among those cases related to SARS-CoV and MERS, a high rate of adverse pregnancy outcomes including abortion, preterm birth, fetal growth restriction, and maternal or neonatal death had been presented (Wong et al., 2004; Alfaraj et al., 2019; Rasmussen et al., 2020). Considering the resemblance to SARS-CoV and MERS, researchers proposed that COVID-19 has the potential to result in maternal or perinatal adverse outcomes for pregnant women (Favre et al., 2020). As a matter of fact, until now, increasing cases and studies have been exhibited, recounting occurrences of adverse pregnancy outcomes during COVID-19. Through digging those data, it has been concluded that COVID-19 infection is associated with a higher rate of preterm birth, preeclampsia, cesarean, and perinatal death (Di Mascio et al., 2020; Juan et al., 2020; Rasmussen et al., 2020). However, the mechanism related to those clinical manifestations has not been well elucidated. There are studies claiming a high expression of ACE2 in the endometrium, and its protein abundance increased during decidualization (Chadchan et al., 2020). ACE2 also exists in maternal–fetal interface cells including stromal cells and perivascular cells of decidua, cytotrophoblast (CTB), and syncytiotrophoblast (STB) in placenta with a dynamic fluctuation over time (Li et al., 2020), thus providing a target receptor for SARS-CoV-2 entering endometrial stromal cells and eliciting pathological manifestations in women with COVID-19. Unified with this theory, a study comprising 11 SARS-CoV-2-positive pregnant women reported the presence of SARS-CoV-2 in placental and fetal membrane swabs of three patients (Penfield et al., 2020). Furthermore, localization of SARS-CoV-2 spike protein and RNA was found in the villi and peri-villous fibrin, and infiltration of macrophages was reported in placental sections (Hosier et al., 2020).

More conjectures about the pathogenesis of adverse pregnancy outcomes are based on the immunological status of pregnant women, picturing as inflammation alteration and cytokine storm related to infection. Pregnancy is an immunological condition in which the semi-allogeneic fetus grows in the mother’s uterus. The immunological environment during pregnancy changes as pregnancy proceeds initially through Th1 (pro-inflammatory response) during the first trimester, then changes to Th2 (anti-inflammatory response) by the second trimester, and again alters toward a Th1 phenotype at the end of third trimester concomitant with initiation of parturition (Weetman, 2010; Verma et al., 2020). Break of the immunological status during pregnancy will definitely do harm to successful delivery.

Virtually all organs and biological systems possibly suffer from this new coronavirus infection by either direct virus-targeted damage or indirect effects. As the pandemic rapidly spread, thyroid dysfunction associated with COVID-19 has been gradually reported (Brancatella et al., 2020; Ruggeri et al., 2020; Marazuela et al., 2020). However, there is conflicting evidence regarding the effects of COVID-19 on thyroid function. COVID-19 has been reported to cause subacute thyroiditis manifesting as marked thyrotoxicosis, as in Graves’ disease (Brancatella et al., 2020; Lania et al., 2020; Muller et al., 2020). It has been found that SARS-CoV-2 causes an overactivation of the immune response through different T-cell lymphocytes (Th1/Th2/Th17), which leads to the activation and release of various pro-inflammatory cytokines including interleukins (IL-1–IL-6) and tumor necrosis factor (TNF-α), culminating in a “cytokine storm” (Lania et al., 2020). The shift in the immune balance between Th1 and Th2 in the body toward Th2 is intrinsic to the pathogenesis of Graves’ disease (Kocjan et al., 2000). Moreover, IL-6, which is elevated in the acute phase, is a specific marker of thyrotoxicosis (Lania et al., 2020). On the other hand, in Chinese studies of COVID-19 patients, general reductions in thyroid-stimulating hormone (TSH), total thyroxine (T4), and triiodothyronine (T3) were more consistent with a non-thyroidal disease pattern (Chen et al., 2021). The degree of decrease in TSH levels correlates positively with the clinical severity of COVID-19 (Khoo et al., 2021). As widely recognized, thyroid hormone (TH) acts as a pleiotropic regulator of growth, differentiation, proliferation, and other physiological processes and is required to maintain the metabolic rate and oxygen consumption in almost all tissues (Chen et al., 2013). For pregnant women, more TH is demanded to maintain the hemostasis concentration during gestation due to the physiological change of thyroid economy (Glinoer et al., 2010). Although it is unclear how SARS-CoV-2 virus affects pregnancy given that pregnancy outcome is influenced by TH levels and the mechanism of SARS-CoV-2 invasion of the thyroid has been well documented. Here, we review previously documented changes in SARS-CoV-2-associated thyroid disease and pregnancy and further discuss various potential mechanisms to help clinicians better understand the impact of SARS-CoV-2 on pregnancy and to facilitate diagnosis and rational treatment of COVID-19.

In the first and early part of the second trimester, fetuses entirely rely on maternal supply of TH (Chen et al., 2015). While from the middle of the second trimester and onward, both maternal and fetal original THs are present in the fetus (Chan et al., 2009). Apparently, the process of transplacental TH exchange involves a cascade of events and masses of TH-related proteins, any impaired link or key protein deficiency during the course will contribute to the reduction of TH exchange, further damaging placenta function and fetal development, leading to adverse pregnant events. T3 and T4 exert effects not only in fetal development but also in placenta function (Landers et al., 2009; Li et al., 2010; Chen et al., 2015). It has been concluded that the possible consequences of hypothyroidism during gestation include spontaneous abortion/miscarriage, gestation-induced preeclampsia, placenta abruption, preterm delivery, congenital anomalies, fetal distress in labor, stillbirth or perinatal death, and increased frequency of cesarean sections (Glinoer et al., 2010). As we can see, outcomes caused by insufficient TH during gestation are highly consistent with those happening in the context of SARS-CoV-2 infection. Consistent to our thesis, there has already been a trial of using T3 for the treatment of critically ill patients with COVID-19 infection (Pantos et al., 2020).

## 2 Maternal Thyroid Hormone Disturbance Caused by SARS-CoV-2

### 2.1 SARS-CoV-2 Directly Attacks Thyroid Gland

Assessment of thyroid function for COVID-19 is not recommended by the World Health Organization clinical management guidelines (Piva et al., 2020). So far, studies associating thyroid or THs with COVID-19 have been indeed scarce, and thyroid gland involvement in COVID-19 infection is not yet clearly defined. Yet, as the pandemic keeps progressing, more than one case of subacute thyroiditis caused by SARS-CoV-2 infection have been reported, showing the direct harm of SARS-CoV-2 to the thyroid gland (Brancatella et al., 2020; Ruggeri et al., 2020). The histopathological report of the thyroid gland in patients with SARS-CoV-2 infection has also been published, noting the follicular epithelial cell disruption (Hanley et al., 2020). However, the significance of this histopathological data regarding the thyroid gland in patients with COVID-19 is uncertain. While in cases of SARS, pathology showed follicular cells were remarkably damaged after infection of SARS, followed by thyroid dysfunction and fibrosis after the acute phase (Wei et al., 2007). And clinical evidence also showed the deficiency of THs relying on lab tests (Scappaticcio et al., 2020). Given the similarities SARS-CoV-2 shares with SARS, it is highly possible that newly affected COVID-19 patients are also suffering from similar thyroid impairment, though the extent may be subclinical.

On the other hand, a study indicated that ACE2 expression in the thyroid gland was among the top 10 in all body tissues (Wang et al., 2020). More studies have also confirmed that both ACE2 and TMPRSS2 are highly expressed in the thyroid gland (Lazartigues et al., 2020; Li et al., 2020). Whether the expression of those two proteins can indeed offer targets for virus entry or not still requires more research to define.

### 2.2 SARS-CoV-2 Indirectly Suppresses Thyroid Hormone

**Figure 1:**
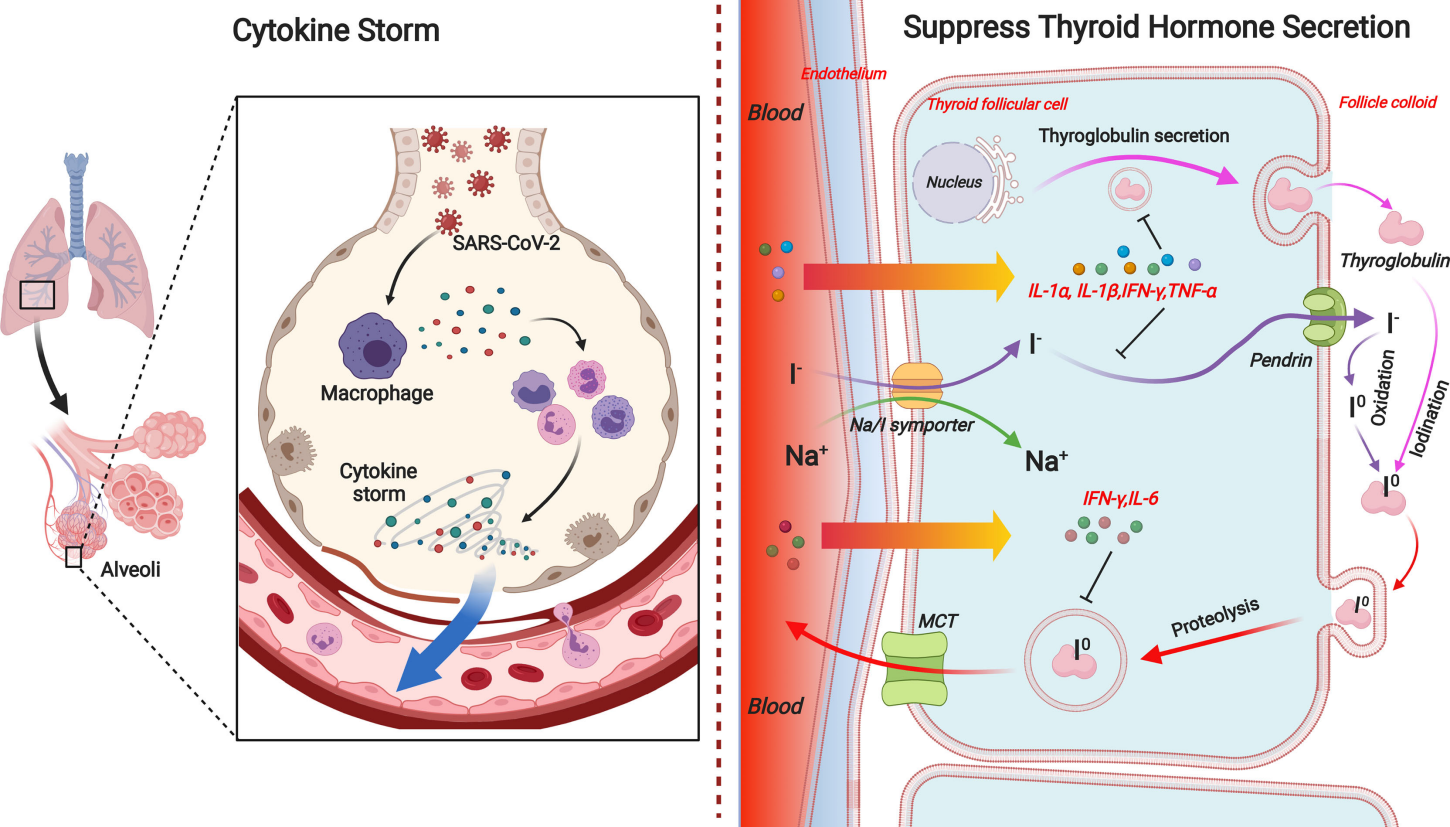
Overview of indirect effects of SARS-CoV-2 on thyroid hormone level in pregnant women. SARS-CoV-2 infection can promote disarrangement of thyroid hormone in pregnant women directly through thyroid gland damage or indirectly through inflammation-induced suppression in diverse links of hypothalamic–pituitary–thyroid axis. Acute inflammation caused by coronavirus infection can respectively reduce TRH and TSH production or release. Pro-inflammatory cytokines, especially IL-1α, IL-1β, IL-6, IFN-γ, and TNF-α, are responsible for diminished iodide uptake, TH secretion, or Tg production by means of individual or collaborative style. On the other hand, the expression and activity of deiodinases in tissues, i.e., D1 and D3 are directly suppressed or inactivated by inflammation or illness. TRH, thyrotropin-releasing hormone; TSH, thyroid-stimulating hormone; TH, thyroid hormone; D1, deiodinase 1; D3, deiodinase 3.

As we all know, SARS-CoV-2 infection appears to induce an acute inflammatory status combined with a mixture storm of cytokines and chemokines, including IL-1α/β, IL-2, IL-6, IL-8, IL-17, IL-10, TNF-α, interferon (IFN)-γ, macrophage colony-stimulating factor (M-CSF), and granulocyte colony-stimulating factor (G-CSF) (Wei et al., 2007; Hanley et al., 2020; Piva et al., 2020). Those cytokines and acute reactive chemokine eruption were also observed in pregnant women (Scappaticcio et al., 2020; Verma et al., 2020). Such pro-inflammatory status after SARS-CoV-2 infection is definitely unfavorable during pregnancy and is responsible for the pathogenesis of non-thyroidal illness syndrome (NTIS). Its effect expands the predominant central downregulation of hypothalamic–pituitary–thyroid (HPT)-axis feedback loop mechanism, enrolling local TH management turbulence. Thus, both maternal systemic and local TH deficiency in placenta can be caused. The possible pathways are concluded below (**Figure 1**).

#### 2.2.1 Deficiency of Central Hypothalamic–Pituitary–Thyroid-Axis Feedback Loop

NTIS is characterized by reduced circulating levels of T3, increased levels of rT3, normal or low serum total concentrations of T4, increased or decreased free T4 (FT4) level, unaltered or inappropriately low serum thyroid-stimulating hormone (TSH), indicating impaired TH conversion, and profoundly altered negative feedback in the pituitary and hypothalamus (Economidou et al., 2011; Farwell, 2013; Hercbergs et al., 2018). It occurs in a variety of non-thyroidal illnesses (NTIs). The condition of NTIS is considered as an adaptive response rather than true hypothyroidism during acute inflammation or critical disease (Mancini et al., 2016).

Notably, Muller et al. (2020) found that 15% (13/85) of COVID-19 patients admitted to high intensity of care units (HICUs) had atypical thyroiditis, which is recognized as a form of subacute thyroiditis without neck pain. Those patients are characterized by low concentrations of TSH and free T3 (FT3) along with normal or elevated concentrations of FT4. Depending on that study, this ‘atypical thyroiditis’ is more frequently appeared in women, which points to the gender disparity in immune status. Recent large cohort study including completed thyroid function tests also confirmed coronavirus disease 2019 associated with a lower thyrotropin and FT4, but no significant sign of thyrotoxicosis was defined (Khoo et al., 2021). Similarly, according to documents from SARS outbreak in 2003, it has been reported that serum T3, T4, and TSH were all lower in patients with SARS as compared to controls during both the acute and convalescent phases. This could simply imply an underlying NTIS (Marazuela et al., 2020). The synchronic decrease of TSH and T4 suggests impaired feedback loop of HPT axis. Later, in human autopsy, decreased postmortem TRH gene and TRH mRNA expression were observed in the hypothalamic paraventricular nucleus (PVN) of patients with NTIS, suggesting central downregulation of the HPT axis (Fliers et al., 1997). Namely, even though less T4 circulates in peripheral blood, the hypothalamic in NTIS patients cannot effectively respond to the feedback to trigger more TH production and release. Similarly, acute inflammation is found to contribute to remarkable downregulation of hypothalamic TRH expression (Kakucska et al., 1994) and pituitary TSHβ mRNA expression (Fekete et al., 2004; Mebis et al., 2009), which indicates that acute inflammation is capable of inducing NTIS and both hypothalamic and pituitary are pinned down under such circumstance.

#### 2.2.2 Suppressed Thyroid Hormone Synthesis and Release

**Figure 2:**
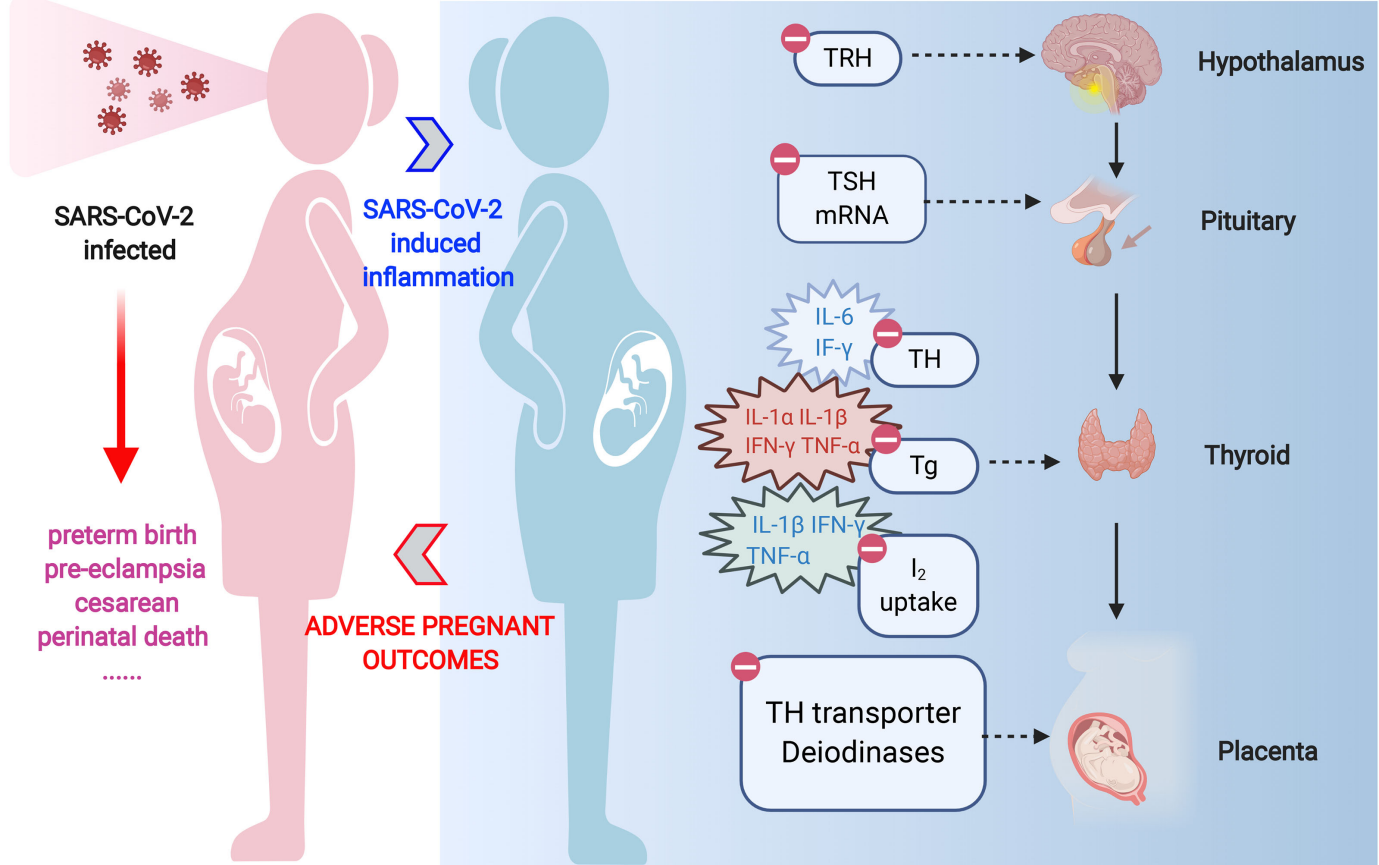
The effects of pro-inflammatory cytokines on TH synthesis pathway in the thyroid. During the process of TH production and release, IL-1α and IL-1β inhibit the TSH-induced Tg mRNA expression and Tg release in human cultured thyrocytes. IL-1β is also responsible for the impairment of basal and TSH-stimulated uptake of iodide by the NIS in porcine thyroid follicle. IL-6 inhibits TPO mRNA expression and T3 secretions. IFN-γ inhibits TSH-induced Tg mRNA expression, Tg and TH secretion, and TSH-induced TPO expression. Besides, TSH-induced increase in NIS expression is eliminated by IFN-γ in rat FTRL-5 cells. TNF-α is known to downregulate Tg production and release in cultured thyrocytes. TNF-α also inhibits NIS expression in rat FTRL-5 cells. TH, thyroid hormone; Tg, thyroglobulin; NIS, natrium/iodide symporter; TPO, thyroperoxidase.

Afterward, the experimental induction of NTIS in rodents resulted in synchronous changes in hypothalamic, pituitary, and peripheral TH metabolism (Boelen et al., 2004), arguing that NTIS is more than a central-level downregulation feedback loop of HPT axis but also a combination of local organ derangement. It has been documented that pro-inflammatory cytokines, either alone or synergistically, are able to downregulate various components of the TH synthesis pathway in the thyroid, consequently leading to decreased secretion of T4 and T3 (Bartalena et al., 1998). Among those associated cytokines, IL-1α, IL-1β, IL-6, IFN-γ, and TNF-α are most frequently mentioned (**Figure 2**).

IL-1α and IL-1β inhibit the TSH-induced thyroglobulin (Tg) mRNA expression and Tg release in human cultured thyrocytes (Krogh Rasmussen et al., 1988). On the other hand, IL-1β is also responsible for the impairment of basal and TSH-stimulated uptake of iodide by the natrium/iodide symporter (NIS) in porcine thyroid follicle (Nolte et al., 1994).

It is demonstrated that IL-6 was found to be negatively correlated with serum T3 concentrations in hospitalized patients (Boelen et al., 1993). Such manifestation can be partly explained by the reason that IL-6 inhibits the TSH- and cathelicidin antimicrobial peptide (cAMP)-induced increase in thyroid peroxidase (TPO) mRNA expression and T3 secretions (Tominaga et al., 1991). Apart from that, IL-6 induces oxidative stress (OS), so that a unifying mechanism might be that cytokine-induced OS alters secondarily the expression and activity of deiodinases (Marsili et al., 2011).

IFN-γ, as one of the cytokines mainly involved in antiviral and antibacterial responses, poses multiple threats on human thyrocytes. It inhibits TSH-induced TH and Tg secretion (Nagayama et al., 1987) and Tg mRNA expression (Sato et al., 1990), TSH-induced TPO expression (Ashizawa et al., 1989), and the TSH- and cAMP-induced upregulation of TSH receptors on the thyrocyte (Nishikawa et al., 1993). Besides, TSH-induced increase in NIS expression is inhibited by IFN-γ in rat FTRL-5 cells, which further results in diminished iodide uptake and subsequent TH synthesis (Ajjan et al., 1998). TNF-α is known to inhibit the TSH-induced cAMP response and Tg production (Deuss et al., 1992) and release (Poth et al., 1991; Rasmussen et al., 1994) in cultured thyrocytes. TNF-α also inhibits NIS expression in rat FTRL-5 cells (Ajjan et al., 1998).

#### 2.2.3 Dysfunction of Thyroid Hormone Transporter and Deiodinases

Apart from the systemic deficiency of THs as a result of central depression and decreased TH synthesis, local regulation and conversion of THs also encounter alteration in the context of inflammation.

Cellular entry of TH is necessary before intracellular conversion of TH by deiodinases and binding to the nuclear thyroid hormone receptor (TR) can take place. TH transporters, monocarboxylate transporter-10 (MCT10), and organic anion transporting polypeptide-4C1 have been presented to be altered in illness or acute inflammation, but the underlying function is still elusive (de Vries et al., 2015). Whether other TH transporters are affected by inflammation still need more investigation.

Another pathogenesis for inflammation causing TH derangement is through influence on deiodinases, leading to TH production and degradation imbalance. There are three types of deiodinases, D1 and D2 are T3-producing enzymes while D3 inactivates T4 and T3. The expression and activity levels of all three deiodinases are likely to be altered during illness or inflammation, in divergent ways, depending on their locations in specific tissues or organs and the severity of illness (Yu and Koenig, 2000; Yu and Koenig, 2006; de Vries et al., 2015). Notably, cytokines are able to suppress the activation of D1 and D2, thus inhibiting T3 generation (Wajner et al., 2011). It has been testified that women in early pregnancy infected by COVID-19 had a higher concentration of FT3 and a lower concentration of FT4 in comparison to those normal ones (Lin et al., 2020). Such phenomenon can be partly explained by the disorder of TH transition and balance.

To sum up, inflammation, together with cytokines triggered by SARS-CoV-2 infection, can negatively impact the maternal TH in the manner of disrupting central feedback loop, suppressing TH synthesis and inhibiting TH transport and conversion, ultimately leading to decreased secretion of T4 and T3.

## 3 Effects of Thyroid Hormone Disturbance Induced by SARS-CoV-2 on Pregnant Women

### 3.1 Non-Thyroidal Illness Syndrome Induces Greater Risk During SARS-CoV-2 Infection

The structural protein of the plasma membrane, integrin αvβ3, is generally expressed and activated in rapidly dividing cells and tumor cells (Ferretti et al., 2007; Davis et al., 2011). The majority of the integrin heterodimeric protein is extracellular and is involved in intercellular binding and in binding to extracellular matrix proteins (Xiong et al., 2007). But this integrin also has a cell surface small molecule receptor for TH and its derivative, tetraiodothyroacetic acid (tetrac) (Davis et al., 2014; Davis et al., 2020). *Via* this receptor, T4 and T3 can activate both the extracellular signal-regulated kinase (ERK)1/2 and phosphatidylinositol 3-kinase (PI3K) pathways (De Vito et al., 2011), subsequently leading to protein trafficking, angiogenesis, and tumor cell proliferation. In contrast, tetrac, a naturally occurring analog of T4, inhibits the binding of both T4 and T3, blocking angiogenesis induced by TH (Davis et al., 2004; Davis et al., 2015). What is notable is that CTBs in human placenta share similarities with neoplastic cells for their proliferative capacities. For that, it is not surprising that CTBs, like endothelial cells during angiogenesis, express αvβ3 (Ferretti et al., 2007). With the aid of TH, this integrin facilitates the invasion, migration of trophoblasts (Ferretti et al., 2007), and brain angiogenesis in the embryo (Davis et al., 2015).

Recently, it has been suggested that integrin αvβ3 is highly possible to be enrolled in the process of SARS-CoV-2 virus uptake (Davis et al., 2020). As widely acknowledged, SARS-CoV-2 is thought primarily to depend on ACE2 (Shang et al., 2020) for entry and the serine protease TMPRSS2 for S protein priming (Hoffmann et al., 2020). While SARS-CoV-2 sequencing analysis revealed a conserved RGD (Arg-Gly-Asp) motif (Sigrist et al., 2020), which is the minimal peptide sequence required for binding proteins of the integrin family (Hussein et al., 2015; Sigrist et al., 2020). According to previous studies, host cellular uptake and the replication of another epidemiologically important coronavirus, porcine epidemic diarrhea α-coronavirus (PEDV), have been proven to require integrin αvβ3 (Li et al., 2019). Given that the integrins containing the binding site for RGD peptides are frequently involved in human virus infection (Hussein et al., 2015), αvβ3 is potentially drown into cellular uptake of SARS-CoV-2 (Davis et al., 2020). Referring to the study of Lin et al. (2013), cellular internalization of αvβ3 is driven by the binding of T4 to the integrin, namely, presence of T4 may support cellular virus uptake. At the same time, *via* αvβ3, TH also generates transcription of a number of cytokines and chemokines (Davis et al., 2020). While the elevation of FT4, as part of the NTIS, may enhance the cell surface abundance and uptake of αvβ3 (Shinderman-Maman et al., 2016). This theory puts tissues containing integrin αvβ3, including placenta, into more dangerous circumstances where they will easily become victims for SARS-CoV-2 and become the victim of a cytokine storm.

### 3.2 Thyroid Hormone Disturbance Induces Placenta Dysfunction

During pregnancy, the uterus undergoes a series of changes that results in extensive tissue reorganization, mainly to accommodate the developing fetus (Correia-da-Silva et al., 2004). The placenta is an organ that provides the maternal–fetal interface between mother and fetus, which is responsible for hormone secretion, fetal nourishment, fetal thermoregulation, fetal waste removal, fetal gaseous exchange regulation, and fetal protection from the maternal immune system and xenobiotics (Burton and Fowden, 2015). During the biological process of mammalian placentation, diverse trophoblast populations gradually form. As the first trophoblast phenotype differentiated from precursors, CTBs subsequently yield STBs and extravillous trophoblasts (EVTs) through further proliferation and differentiation. Those trophoblasts collaboratively mediate the establishment of uteroplacental circulation and placenta formation (Ji et al., 2013). Placental dysfunction is the central characteristic of pregnant complications of human pregnancy, and abnormalities in placental formation and physiology are implicated in miscarriage, preeclampsia, and intrauterine growth restriction (IUGR) (Silva et al., 2012). To maintain robust placental function for a healthy pregnancy, a balance between proliferation and apoptosis, further differentiation, together with normal angiogenesis of placenta are indispensable.

TH is vital for a healthy pregnancy and fetal development, playing multifaceted roles in maintaining the normal function of the placenta. It has been found to be intimately associated with placenta hormone secretion, trophoblast proliferation and differentiation, EVT invasiveness, and decidual angiogenesis (Adu-Gyamfi et al., 2020). As clarified above, the infection of SARS-CoV-2 and the following inflammation can trigger TH derangement for pregnant women. That can be a great threat to pregnancy, since high incidences of mal-placentation-mediated pregnancy complications such as preeclampsia, miscarriage, and IUGR have been reported in women with abnormal levels of THs (Korevaar et al., 2017). The underlying mechanisms are concluded as follows.

#### 3.2.1 Disturbance of Trophoblast Proliferation and Differentiation

CTB cell fusion and hormone secretion indicate the differentiation of CTB to STB (Ji et al., 2013). Treating CTB with T3 at optimal concentration led to a significant increase in human chorionic gonadotropin (hCG) secretion (Silva et al., 2012), indicating the involvement of THs in STB formation. Besides, both T3 and T4 are capable of eliciting a stimulatory effect on placenta hormone secretion of human placental lactogen (hPL), estradiol-17 beta, progesterone, and hCG. However, lower doses of T3 or T4 attenuated such stimulatory effects (Adu-Gyamfi et al., 2020). Which means insufficient T3 and T4 supplementation hampers the endocrine secretion of placenta and deters trophoblast differentiation. Hypothyroid rats present a decrease in the placenta thickness, which attributes to a reduction in the proliferation of trophoblast cells and increase in apoptosis (Oki et al., 2004; Shinderman-Maman et al., 2016; Korevaar et al., 2017). This phenomenon may be caused by the downregulation of placental leptin and increased Toll-like receptor (TLR)2 expression promoted by hypothyroidism (Oki et al., 2004; Vissenberg et al., 2015). As a result, TH insufficiency can be one of the reasons for placenta fragileness and dysfunction.

#### 3.2.2 Disturbance of Extravillous Trophoblast Invasiveness

T3 facilitates EVT invasion of the decidua. On one hand, it has been proven that T3 is responsible for increasing the mRNA expression of matrix metalloproteinases 2 (MMP2), matrix metalloproteinases 3 (MMP3), and fetal fibronectin (Oki et al., 2004; Vissenberg et al., 2015), which are the fundamental elements demanded in the normal biological process of EVT invasiveness (Liao et al., 2015; Zhao et al., 2018). On the other hand, T3 has also been found to suppress EVT apoptosis through the downregulation of Fas and the Fas ligand (Laoag-Fernandez et al., 2004). Without enough T3, the migration of EVTs is markedly reduced in hypothyroid pregnancies (Silva et al., 2014).

#### 3.2.3 Disturbance of Angiogenesis

L-thyroxine induces the gene expression of placental growth factor (PGF) and vascular endothelial growth factor (VEGF) (Silva et al., 2015), which, in early gestation, are considered as the dominant pro-angiogenic factors involved in the vascular development of the maternal–fetus interface (Adu-Gyamfi et al., 2020). In different periods of gestation, decidual cells respond distinctively to T3 as increasing the secretion of vascular endothelial growth factor-A (VEGFA) and angiopoietin-2 (ANGPT2) in the first trimester while increasing angiogenin (ANG) secretion in the second trimester (Vasilopoulou et al., 2014). Researchers had detected a remarkable reduction in the placental expression of VEGF (Silva et al., 2012; Silva et al., 2014) along with increment in placental vascular resistance (Barjaktarovic et al., 2017) in hypothyroid status. Consistent with those findings, both dilation of the maternal venous sinuses in the placental labyrinth (Silva et al., 2012) and reduction in the size of the decidua (Adu-Gyamfi et al., 2020) have also been observed possibly as the consequences of impaired angiogenesis and spiral arteries’ remodeling. Given those solid proofs, abnormal placental TH supplement has a great tendency to affect placental vascularity, which might account for the high incidence of preeclampsia and miscarriage reported among hypothyroid women (Landers and Richard, 2017).

#### 3.2.4 Alteration of Immune Status

As mentioned above, a successful pregnancy requires comparatively suppressive modulation of the immune system to ensure the coexistence of mother and fetus. And maternal immune status fluctuates as the gestation advances. Decidua is responsible for releasing inflammatory mediators during pregnancy (Toder et al., 2003; Koga et al., 2009; Hu and Cross, 2010), and abnormal alterations of such molecules have been reported to be associated with miscarriage (Vassiliadis et al., 1998) and preeclampsia (Valencia-Ortega et al., 2019). In hypothyroid conditions, there is a compromise in the establishment of an anti-inflammatory environment in the placenta, which is evidenced by a decrease in placental IL-10, leptin, and nitric-oxide synthase 2 (NOS_2) expression (Silva et al., 2014). Similarly, hypothyroid women exhibit reduced expression of IL-4 and IL-10 in the decidua (Twig et al., 2012). The release of inflammatory cytokines at the fetal–maternal interface partly depends on the activation of TLRs. Interestingly, placental TLR expression is also affected by THs, as evidenced by the reported increase in TLR2 levels and a reduction in TLR4 levels in the placenta of hypothyroid pregnancies (Silva et al., 2014), similarly leading to a reduction in the gene and/or protein expression of the anti-inflammatory cytokines IL-10 and NOS2. After being infected by SARS-CoV-2, there will be a tendency for establishing a pro-inflammatory response against viruses, adding up with TH turbulence, and pregnant women definitely will face greater risks.

## 4 Dysfunction of Thyroid Hormone Transporter and Deiodinase and Related Pregnant Complications

TH enters and exit the placental cells through six TH membrane transporters: large amino acid transporter-1 (LAT1), LAT2, organic anion transporting polypeptide-1A2 (OATP1A2), OATP4A1, monocarboxylate transporter-8 (MCT8), and MCT10 (Adu-Gyamfi et al., 2020). As mentioned above, TH transporters may be altered during inflammation, such as MTC-10. Interestingly, in severe IUGR villous placentas, MCT8 expression is significantly increased, while MCT10 expression is significantly decreased (Loubiere et al., 2010). Although MCT-10 may not serve as the most essential TH transporter in the placenta, at least it suggests that the abnormalities of TH transporters during inflammation are not purely innocent in pregnant complications.

Within the placenta, TH is mostly acted on by D2 and D3. As clarified above, during inflammation, cytokines are able to suppress D2 activation, thus inhibiting T3 generation. That means inflammation caused by SARS-CoV-2 can become a risk factor for pregnancy, leading to lack of T3 in placenta and fetus. Furthermore, the main regulator of TH homeostasis in the placenta is D3 (Adu-Gyamfi et al., 2020), which protects the fetus from an overexposure to T3. D3 activity possibly alters in the context of inflammation. During acute and chronic inflammation and during sepsis, liver Dio3 mRNA expression and activity levels are decreased (de Vries et al., 2015). If it is a similar case within the placenta during SARS-CoV-2-induced inflammation, that can negatively affect fetus because of overexposure to T3. Notably, there is an observation suggesting a possible blunting of D3 activity in preeclampsia (Kurlak et al., 2013). Abnormal upregulation of the placental D3 gene is a potential contributor to fetal hypothyroidism because the more D3 remains active, the less active TH will be transferred to the fetus (Wilcoxon and Redei, 2004). The upregulation of D3 is rarely seen and usually occurs in conditions of prolonged critical illness or inflammation (de Vries et al., 2015), but further study indicated that the prolonged reduction of food intake during illness may be the dominant trigger for D3 upregulation (de Vries et al., 2014). Hence, it may serve as a reminder that during SARS-CoV-2 infection and treatment, pregnant women should better avoid fasting for too long.

## 5 New Insights Into the Management of Pregnant Women During the COVID-19 Pandemic

Significant physiological changes in the THs of pregnant women occur during pregnancy (Fan et al., 2019). Around the fifth or sixth week of pregnancy, although the fetal thyroid is beginning to develop, the fetus is not yet able to synthesize its own THs at this time (Patel et al., 2011). Therefore, the fetal TH required for normal neurodevelopment comes exclusively from the mother. Current studies suggest that SARS-CoV-2 infection alters thyroid function in early pregnancy and that there is an increased risk of adverse pregnancy outcomes (Lin et al., 2020). The relationship between ACE2 expression levels during SARS-CoV-2 infection is intricate, with high ACE2 expression favoring the entry of SARS-CoV-2 host cells, while reduced ACE2 expression following infection may lead to severe disease (Ni et al., 2020). THs play a key role in determining ACE and ACE2 expression in plasma and different tissues, which in turn may play a role in the severity of SARS-CoV-2 infection and disease (Kumari et al., 2020). Therefore, TH levels in COVID-19 pregnant women are of interest. However, further research is needed to determine whether such monitoring and treatment will lead to safe and effective outcomes.

Vaccines are currently one of the most promising preventive measures against COVID-19 (Iqbal Yatoo et al., 2020). Vaccination during pregnancy is a promising strategy to protect mothers and newborns from SARS-CoV-2 infection (Saxena et al., 2020). However, live or live attenuated vaccines may not be safe because of the risk of disease in the immune-regulated gestational state (Kumar et al., 2021). Recently, there have been some reports of thyroid problems following vaccination (Joob and Wiwanitkit, 2021; Vera-Lastra et al., 2021). For example, Vera-Lastra et al. (Vera-Lastra et al., 2021) noted that SARS-CoV-2 vaccination may induce hyperthyroidism. Furthermore, adjuvants may cause alterations in the immune system and cause thyroid problems. In terms of pathophysiology, COVID-19 vaccination causes an increase in blood viscosity (Joob and Wiwanitkit, 2021). High blood viscosity is an important factor in abnormally high TH levels (Tamagna et al., 1979). On the other hand, inactivated or nucleic acid vaccines may be safer because there is no risk of disease from this kind of vaccine (Vora et al., 2020). Overall, we considered that the selection of a reasonable COVID-19 vaccine is essential to induce a balanced humoral and cell-mediated immune response without overactivating the maternal immune system (Vora et al., 2020). An inappropriate vaccine will lead to TH disorders, thereby inducing an adverse pregnancy.

## 6 Conclusion

We provide a plausible overview relating to COVID-19, TH, and pregnancy, elucidating the possible mechanism that COVID-19 would give rise to adverse pregnancy outcomes. SARS-CoV-2 causes an overactivation of the immune response and culminates in a “cytokine storm,” which, on one hand, leads to a disturbance in maternal TH, and on the other hand, causes dysfunction of TH transporter and deiodinase in placenta. The overall TH disturbance in pregnant women eventually induces placenta dysfunction, including disturbance of EVT invasiveness and angiogenesis, and alteration of immune status. Therefore, physicians should raise alertness on TH abnormality when treating pregnant COVID-19 patients.

## Author Contributions

Conceptualization: PH and SC. Resources: QS, PC, MW, and BW. Data curation: PC, MW, and BW. Writing: QS, MW, and PH. Supervision: PH and SC. Funding acquisition: PH and SC. TH’s contribution to this article is in the manuscript writing. All authors contributed to the article and approved the submitted version.

## Funding

This work was supported by grants from the National Natural Science Foundation of China (81974423, 81902729), the Key Research and Development Programme of Hunan Province of China (2019SK2031), the Natural Science Foundation of Hunan Province (2020JJ5904), and China Postdoctoral Science Foundation (2020M672517, 2021T140749).

## Conflict of Interest

The authors declare that the research was conducted in the absence of any commercial or financial relationships that could be construed as a potential conflict of interest.

## Publisher’s Note

All claims expressed in this article are solely those of the authors and do not necessarily represent those of their affiliated organizations, or those of the publisher, the editors and the reviewers. Any product that may be evaluated in this article, or claim that may be made by its manufacturer, is not guaranteed or endorsed by the publisher.

## Abbreviations

ACE2, angiotensin II-converting enzyme 2; ANG, angiogenin; ANGPT2, angiopoietin-2; CTB, cytotrophoblast; EVT, extravillous trophoblast; hCG, human chorionic gonadotropin; hPL, human placental lactogen; HPT axis, hypothalamic–pituitary–thyroid axis; IUGR, intrauterine growth restriction; LAT, Linker for activation of T cells; MCT, monocarboxylate transporter; MERS, Middle East respiratory syndrome; NIS, natrium/iodide symporter; NTIS, non-thyroidal illness syndrome; OATP, organic anion-transporting polypeptide; PEDV, porcine epidemic diarrhea α-coronavirus; PVN, paraventricular nucleus; RGD, Arg-Gly-Asp; STB, syncytiotrophoblast; T3, triiodothyronine; T4, thyroxine; Tg, thyroglobulin; TH, thyroid hormone; TSH, thyroid-stimulating hormone; VEGF, vascular endothelial growth factor.
